# Xeroderma pigmentosum and acute myeloid leukemia: a case report 

**DOI:** 10.1186/s13256-021-02969-1

**Published:** 2021-08-26

**Authors:** H. Bencharef, M. Lamchahab, D. Dassouli, S. Sraidi, B. Guennoun, N. Hda, B. Oukkache, A. Quessar

**Affiliations:** 1 Hematology and Oncology Pediatric Department, Hospital August 20, 1953, 6 Rue Lahcen Al Arjoun, Casablanca, Morocco; 2 Faculty of Medicine and Pharmacy of Casablanca, Hassan II University, Casablanca, Morocco; 3 Hematology Laboratory, IBN ROCHD University Hospital Center, 1, Rue des Hôpitaux, Casablanca, Morocco; 4 Analysis Laboratory of HDA (Medical Biology and Cytogenetics), Rue Tarik Bnou Ziad, Quartier, Les Hôpitaux, Casablanca, Morocco; 5 Bio-Medical Studies Department, Faculty of Medicine and Pharmacy of Casablanca, Hassan II University of Casablanca, UH2C 19 Rue Tarik Ibnou Ziad, Casablanca, Morocco

**Keywords:** Xeroderma pigmentosum, Acute myeloblastic leukemia, Complex karyotype, Chemotherapy

## Abstract

**Background:**

Xeroderma pigmentosum is a rare inherited disease characterized by extreme hypersensitivity to ultraviolet rays and predisposing to cutaneous malignancies that can appear in childhood. These manifestations are often associated with ocular lesions and sometimes with neurological disorders. The association of xeroderma pigmentosum with internal neoplasms such as acute myeloblastic leukemia is not reported with great frequency, which confirms the rarity of this occurrence.

**Case report:**

A 26-year-old Moroccan women, xeroderma pigmentosum patient, was diagnosed with acute myeloblastic leukemia with a complex karyotype. Due to the adverse risk of the xeroderma pigmentosum association with acute myeloblastic leukemia and the profile of acute myeloblastic leukemia with complex karyotype and monosomy 7, which constitute factors of poor prognosis, as well as the absence of studies conceding the tolerance of the chemotherapy by patients suffering from xeroderma pigmentosum, our patient was put under low-dose cytarabine protocol with granulocyte colony-stimulating factor. Unfortunately, she died on the tenth day of chemotherapy by acute pulmonary edema of cardiogenic pace complicated by tamponade.

**Conclusion:**

According to reports, it is the second case showing association of xeroderma pigmentosum with acute myeloblastic leukemia. The management of these patients remains a challenge. Studies focusing on xeroderma pigmentosum patients developing hematological malignancies are necessary to better understand the most appropriate strategies and precautions for this specific case.

## Introduction

Xeroderma pigmentosum (XP) is a rare inherited disease described initially by Kaposi in 1870. This disease is characterized by extreme hypersensitivity to ultraviolet rays predisposing to cutaneous malignancies that can appear in childhood. These manifestations are often associated with ocular lesions that can lead to blindness and sometimes with neurological disorders [1, 2].

Different genetic groups correspond to clinical sites of varying severity. Despite a high pathogenic understanding, the evolution is often fatal in the absence of adequate preventive measures.

XP is inherited as an autosomal recessive mode. It has been described in all populations [3], with an estimated prevalence of 1/300,000 in the USA and Europe. It is less rare in Japan, where the prevalence is estimated at 1/100,000. In India, the incidence has not been studied so far but is believed to be less than in Western countries [4, 5].

However, it is still relatively frequent in certain regions with high levels of consanguinity and relatively large families, such as in the Middle East and the Maghreb. The incidence is estimated at 1/10,000 in Tunisia [6, 7].

The geographical distribution of the different genetic groups is heterogeneous, with, however, a predominance of certain forms depending on the region: group C is the most frequently reported in the Mediterranean countries and groups A and F in Japan.

In Morocco, studies of molecular epidemiology have shown that two mutations predominate in our population. These are the recurrent mutations c.1643_1644delTG of the XPC gene, followed by the c.682C> T mutation of the XPA gene [8].

There is no sex predilection. Pathophysiology of this disorder is a defect in genes within the nucleotide excision repair system for the first seven genetic groups (A–G), and an abnormality in transcription groups for the eighth group (xeroderma pigmentosum variant, XPV).

Normal individuals harboring XP polymorphisms are at increased risk for developing acute lymphoblastic leukemia and acute myeloid leukemia (AML) [9].

AML in XP is very rare. We present here an interesting case of XP associated with acute myeloid leukemia.

## Case report

A 26-year-old Moroccan woman was born of a consanguineous marriage (first degree). One of four siblings, she was diagnosed with XP at the age of 3 months.

The diagnosis of XP was made based on the family history, because the patient had a brother who died at the age of 4 years and a sister who died at the age of 27 years, by metastases of melanomas secondary to XP.

Since childhood, she presented dermatological symptoms, photosensitivity, itching over the face, and hyperpigmentation that progressed with age.

Therefore, erythematous scaly plaques with pigmented macules were seen over the areas exposed to the sun. She never developed neurological manifestations.

The patient was admitted to our service because of bone marrow failure syndrome with anemic symptoms including paleness, vertigo, tachycardia, and exercise dyspnea, as well as hemorrhagic symptoms including gingivorrhages of low abundance and fever for a 1 month.

**Fig. 1:**
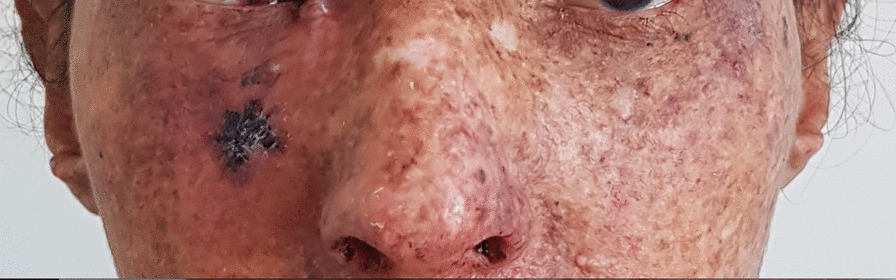
Dyschromia characteristic of xeroderma pigmentosum in photoexposed zones

Physical examination showed that the patient had bad general condition, Eastern Cooperative Oncology Group (ECOG) at 1, stable hemodynamically and in terms of respiration, tachycardia at 120  beats/minute, afebrile with ectropion of the right eye and skin lesions including multiple lentigines and papular/ulcerated lesions suggestive of cutaneous malignancies (Fig. 1), poor oral condition without gingival hypertrophy, and no adenopathy or organomegaly. Cardiac auscultation and neurological examination were normal.

Complete blood count showed pancytopenia (white blood cell count 2.26 g/L, hemoglobin 4.2 g/dL, and platelets 10 g/L).

Peripheral smear revealed the presence of 70% blast cells.

**Fig. 2:**
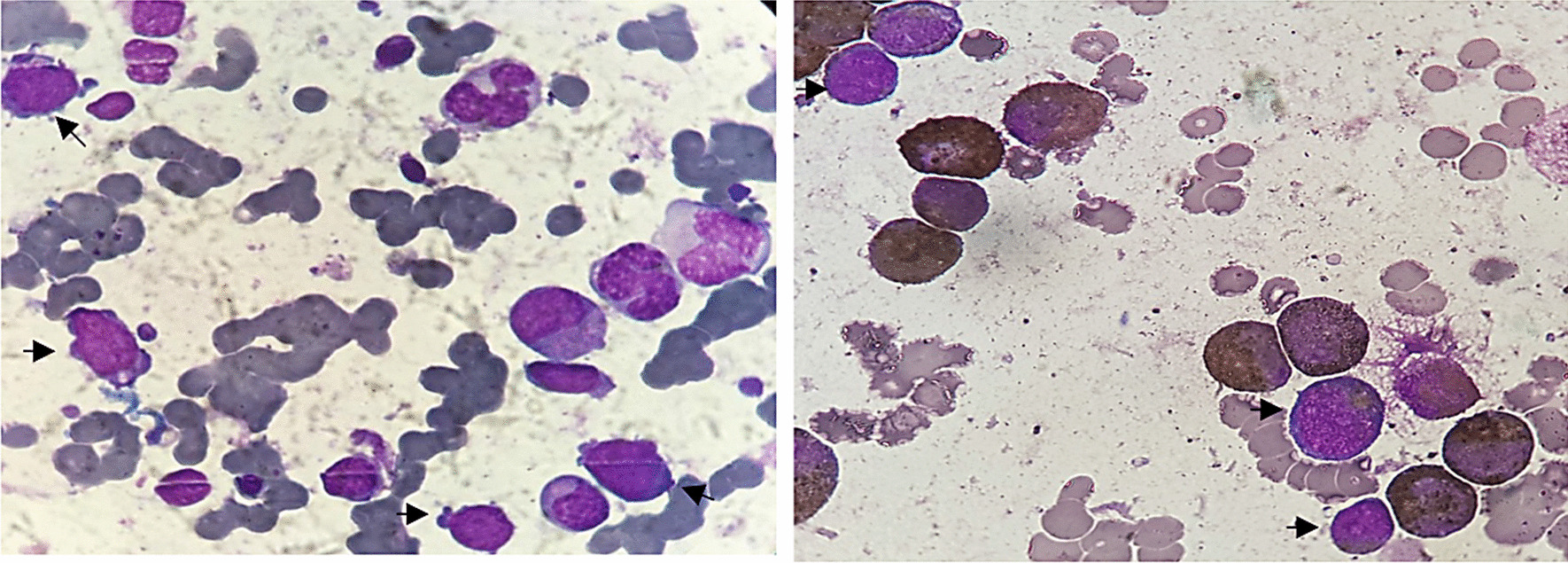
Bone marrow aspiration showing infiltration by morphologically undifferentiated blasts with positive MPO (arrows show blast cells positive for MPO)

On bone marrow aspiration, infiltration by 25–30% morphologically undifferentiated blasts was seen with positive myeloperoxidase (MPO) (Fig. 2), without any dysplastic features.

Flow cytometry revealed expression of a low CD45 blast population (34%) HLA DR+ CD33+, CD117+, CD13+, CD34+, and lymphoid markers were negative, compatible with the diagnosis of acute myeloblastic leukemia.

**Fig. 3:**
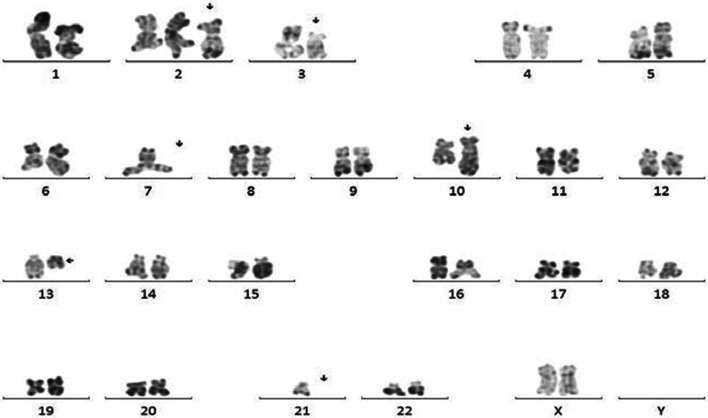
Results of chromosome banding analysis: 46, XX,del(3)(p13?), der 10 t(10,?) (q26, ?) der 13 (13,?) [2]/46, idem, +2, -21[2]/45, idem, -7[25] /69~71idem x2 [8]

Chromosome banding analysis showed a complex karyotype with numerous numerical and structural chromosomal aberrations (Fig. 3): initial clone with derivative of chromosome 10, a derivative of chromosome 13, a deletion of (3) (p13?); a first subclone with a trisomy 2 and monosomy 21; a second subclone with monosomy 7; a third hyperdiploid subclone with a deletion of (3) (p13) in double and a clone without abnormalities. Due to lack of resources, fluorescence *in situ* hybridization (FISH) and polymerase chain reaction (PCR) studies were not performed.

Viral serologies (viral hepatitis B and C, and HIV) were normal as well as cardiac ultrasound with good ejection fraction.

Due to the adverse risk of the XP association with AML and the profile of AML with complex karyotype and monosomy 7, which constitute factors of poor prognosis, as well as the absence of the studies conceding the tolerance of the chemotherapy by patients suffering from XP, our patient was put under low-dose cytarabine protocol with cytarabine 10 mg/m^2^/day (d1–d10) and granulocyte colony-stimulating factor (G-CSF): 5 μg/kg/day (d1–d10).

On the tenth day of chemotherapy, the patient exhibited deep pancytopenia with a white blood cell count of 1 g/L, hemoglobin 4 g/dL, and platelets < 10 g/L. On clinical examination, the patient was dyspneic with auscultation of crepitant rales at the level of both lung fields. During the previous 24 hours, she had only received one platelet transfusion since the request for red blood cells was still being processed by the transfusion center of Casablanca.

Chest X-ray revealed diffuse confluent opacity, electrocardiogram indicated supraventricular tachycardia, and echocardiography showed pericardial effusion of low abundance. The patient was placed under water restriction and diuretic bolus; the evolution was marked by the persistence of the dyspnea. A second echocardiography was realized objectifying the exaggeration of the pericardial effusion; an evacuating puncture was indicated, but the situation was aggravated by the occurrence of desaturation when wearing high-concentration mask, hypotension, and cardiorespiratory arrest. The patient died following acute pulmonary edema of cardiogenic pace complicated by tamponade.

## Discussion

XP is a rare form of general dermatosis caused by defects in the normal repair of DNA damaged by exposure to sunlight. It has been reported to be common with consanguineous marriages [10] because it is transmitted in an autosomal recessive mode. This explains its higher frequency in Maghreb countries, including Morocco, marked by a high rate of consanguinity (15.25%) and nuclear family structure [11].

The incidence and age at presentation vary, ranging from early infancy to adulthood.

There are eight complementation groups of XP (XP-A to G) caused by defective nucleotide excision repair, corresponding to mutations in the genes encoding XPA, XPB, XPC, XPD, XPE, XPF, XPG, and XP dominant. This genetic heterogeneity is responsible for the diversity of clinical situations [12, 13].

The severe form is characterized by an early onset, before the age of 1 year, by a cutaneous erythema associated with intense photophobia. Malignant skin tumors appear in early childhood. Survival is uncommon, with patients usually dying before the age of 15 years. This form corresponds to Maghrebian XPC whose mutation is “V548A fs XR72” [14]. The mild form is characterized by having more severe onset (age > 3 years), erythema and photophobia being rare, dyschromia being late onset, and not being evident until the age of 5 years; malignant skin tumors appear relatively late, around the age of 20 years or older. Neurological manifestations are absent. It corresponds to XPF and XPV [15, 16].

The association of XP with internal neoplasms such as acute myeloblastic leukemia is not reported frequently, confirming both their infrequency and their incidence, which remains 10–20 times higher than in the general population [1].

In the present article, we report the case of a patient suffering from XP type C who developed an acute myeloblastic leukemia with a complex karyotype and monosomy 7. The XPC gene is located in the 3p25.1 chromosomal region [1]. This gene plays an important role in the early steps of global genome nucleotide excision repair (NER), especially in damage recognition, open complex formation, and repair protein complex formation. The link between XPC and cancers of various kinds has been investigated in many studies [17, 18], but there is still no evident association between the different malignancies and XPC. It was even demonstrated in an interesting study in the Romanian population that XPC polymorphisms are not associated with AML [19]. Thus, it is not surprising that there are no further cases in the literature describing the development of leukemia in XPC patients. AML in XPC is very rare.

An American study [20] found no significant association between XPC polymorphisms and disease-free survival of patients with AML in the USA. Despite that, in the Chinese population [21], XPC polymorphisms are important markers for the outcome of patients with AML.

A Tunisian study [22] on the analysis of the link between the NER DNA repair genes polymorphisms and susceptibility to leukemia in the Tunisian population found that XPC polymorphism may be involved in the susceptibility to AML.

The difficulty in our case resides not only in the association of XP and leukemia, but also in the complexity of karyotype with monosomy 7, which constitute poor prognostic factors and can explain the bad tolerance of chemotherapy by our patient.

On the other hand, the role of the XPC DNA repair gene polymorphisms in increased chemotherapy toxicity has been discussed controversially in the literature, but none of the studies included XPC in their analysis [23].

To the best of our knowledge, only a single case of XP type D in association with acute megakaryoblastic leukemia has been reported [23].

It remains unclear whether a mutation in the XPC DNA repair gene in this patient led to the development of acute myeloblastic leukemia or a change of tolerance to chemotherapy. Finally, could our patient not be treated with full doses of AML chemotherapy without encountering unusual toxicity? The management of these patients remains a challenge.

## Conclusion

This is the second reported case of the association of XP and acute myeloblastic leukemia. The management of these patients remains a challenge. Studies focusing on XP patients developing hematological malignancies are necessary to better understand the most appropriate strategies and precautions for this specific case.

## Data Availability

All data generated or analyzed during this study are included in this published article.

## Abbreviations

XP: Xeroderma pigmentosum
AML: Acute myeloblastic leukemia
XPC: Xeroderma pigmentosum type C
HIV: Human immunodeficiency virus
DNA: Deoxyribonucleic acid
